# The application of deep learning in economic analysis and marketing strategy formulation in the tourism industry

**DOI:** 10.1371/journal.pone.0321992

**Published:** 2025-06-06

**Authors:** Jing Zhang, Ming Gao

**Affiliations:** Hospitality Management Department, Tourism College of Zhejiang, Hangzhou, China; University of Naples Federico II: Universita degli Studi di Napoli Federico II, ITALY

## Abstract

The tourism industry is ever-evolving in nature, as it operates in a global marketplace that has become progressively global and offers great potential due to technological advances. The tourism industry faces challenges in accurately forecasting economic impacts and understanding visitor patterns that rapid global changes. Motivated by these needs, this research introduces the Tourism Variational Recurrent Neural Network (TourVaRNN), aiming to enhance the tourism industry by predicting economic impacts and visitor behaviors for effective marketing strategies through advanced Deep Learning (DL) techniques. The research applies a variational recurrent neural network for enhancing tourism demands and model the complex temporal dependencies within tourism data. The proposed TourVaRNN integrates variational autoencoders to capture latent variables representing visitor preferences and spending habits, while recurrent neural networks model complex temporal dependencies in tourism data. Marketing campaigns in the tourism sector can be fine-tuned through visitor segmentation, which seeks to comprehend and classify visitors according to their demographics, preferences, and behaviors. The model employs robust forecasting of economic impacts, visitor spending patterns, and behavior while accounting for uncertainty through variational inference. The implementation uses Python language on a tourism dataset comprising necessary attributes like visitor numbers, days, spending patterns, employment, international tourism samples over a specific region, and a diverse age group analyzed over a year. The proposed method is evaluated in terms of performance metrics such as economic impact assessment, visitor segmentation efficiency, inference time analysis, and budget allocation utilization for effective economic and marketing strategy analysis in the tourism industry. TourVaRNN’s improved segmentation efficiency of 15.7 percent allows for more targeted marketing, increasing engagement with visitors and income. Decisions may be made in real-time, improving operational efficiency in tourism management, thanks to a 17.5% reduction in inference time (to 40 ms). The most efficient use of funds is guaranteed by a 13.4% rise in budget allocation utilization, leading to maximum economic benefits.

## 1 Introduction

The tourism industry can transform by adopting new strategies and innovations to meet evolving traveler/visitor demands. This includes liberalized interest rates, employment opportunities, varying earning levels to adapt to market changes and economic growth [1,2]. Embracing revolutionary developments in the travel industry has transformed worldwide activities, increased operational efficiency, and defined the future of marketing strategy [3]. Evolving tourist behavior and demand forecasting, impacted by tourist activities and visiting patterns, can lead to unpredictable demand patterns. This requires short-term operational decisions and long-term planning to manage their revenue and economic levels effectively [4]. For making strategic planning processes during tourism activities using branding campaigns, hotel feedback improves the efficiency of marketing communication with visitors [5]. The tourism industry emphasizes government response, technology innovation, and visitor importance, generating a global economic order [6].

ML techniques for real estate values analyze the property value changes inference by tourism [7]. Deep learning on tourism businesses refines their marketing strategies and improves services through review-based insights [8]. Research on tourism’s impact on factors like tourist’ arrivals influence direct and indirect employment and identified seasonal impact without offering predictive solutions [9]. The advanced technologies change hotel elements’ operational and marketing strategies for tourist destinations through demand forecasting of individuals in revenue generation [10]. Developing focus related to tourism elements like travellers utilized intelligent tourism services with ML algorithms to recognize region congestion rates maintained a narrow behavioral scope [11].

Tourism demand forecasting establishes nonlinear relationships between tourist arrivals and destination search volumes [12]. In an earlier tourist trend, a trip planner application investigated the seasonal visitor behavior and evaluated strategies to encourage off-peak travel. The results demonstrate that Expenditure is higher during off-peak seasons and that events are more effective than applications [13]. The tourism industry’s dynamic nature presents significant chances for applying DL techniques to support better economic analysis and strategic planning for market trends [14]. Earlier studies employed DL to predict sentiments in tourism reviews, improve customer satisfaction analysis, enhance economic insights, and optimize hospitality marketing strategies [15]. The application of DL techniques mainly enhances the accuracy of forecasting tourism volume, which was evaluated using Hong Kong inbound arrivals data using a bi-directional gated recurrent unit (Bi-GRU) [16]. The development of the international tourism market enhances the potential of the sustainable condition of the world economy and its associated national economies, promotes travellers coming from different regions of the world and provides opportunities related to employment [17]. The significance of DL techniques emerges to overlook the diverse solutions of segmenting the customers and delivering precise forecasts to real-time analysis of visitor satisfaction through spending patterns in marketing [18].

### Problem statement

Traditional tourism forecasting models, such as deep neural networks, long-term term models, and others, struggle with capturing complex temporal dependencies, especially in highly seasonal and volatile demand environments. Existing forecasting models fail to adapt to sudden shifts like economic crises, leading to forecast errors exceeding 20% in some cases. Standard LSTMs overfit short-term patterns, limiting their generalization to long-term trends. Hence, the introduced TourVaRNN addresses these gaps using variational inference, enhancing adaptability and accuracy in dynamic tourism markets.

### Purpose of the research and its novelty

The recent works of the tourism demand forecasting model’s reliance on less sophisticated methods often misallocates marketing resources, leading to a failure to capitalize on market opportunities. By developing a more accurate predictive model like TourVaRNN, this research seeks to enable tourism businesses to make more informed decisions regarding budget allocation, campaign targeting, and service offerings. The research novelty lies in the TourVaRNN model, which integrates VAE and RNN. Enhancing visitor segmentation by analyzing demographics and spending patterns enables more precise economic impact assessments and optimized budget allocation. The model’s real-time capabilities improve marketing campaign efficiency and support data-driven decision-making.

The innovations of this research include:

i) To accurately forecast economic impacts and visitor behaviors using Tourism Variational Recurrent Neural Network (TourVaRNN), formulate marketing strategies.
ii) To uncover visitors’ complete range of economic value in tourism, including influence on employment and expenditures.
iii) To provide a foundation for market strategy formulation with a detailed analysis of traveler activities and the dynamic nature of economics.

The following sections discuss the arrangement of the research article.

A concise summary of recent literature works in the field of the tourism industry is discussed in Section 2. Section 3 talks about the advanced DL algorithm employed in this research as the TourVaRNN algorithm for analyzing the visitor patterns and its impact on economic growth and marketing strategy formulation. Section 4 delves into the detailed analysis of tourism data employed in this research and the performance comparison study results implementation. Section 5 concludes the research work and the future scope.

## 2 Related works

### 2.1 Tourism experience and behavioral insights

The correlations between service quality parameters and MTE dimensions were analyzed by Polyakova et al. [19] using a structural equation modeling (SEM) technique. Eight hundred seventy-eight swimmers from 10 different Oceanman events across various countries contributed their responses to the dataset. Hedonic and local cultural characteristics were the most robust predictors of MTEs’ effects on word-of-mouth behavior, as validated by the results. The inability to generalize to different event types was a significant weakness caused by the study’s narrow geographic and event-specific emphasis.

In contrast, Neshat et al. [20] suggested a Deep Neural Network (DNN) model to predict long-term policies attracting tourists to developing nation’s economies after the pandemic. The model promoted domestic tourism and disaster preparedness as the best action based on analyzing crisis features and historical data. The case study demonstrated that it can help with tourist management decision-making, concrete policy suggestions, and prediction of policy outcomes. The model’s forecast accuracy and usefulness in quickly changing settings may be affected by its dependence on historical evidence and predictions about foreseeable crisis dynamics.

### 2.2 Tourism demand forecasting and regional trends

Similarly, Pan et al. [21] analyzed tourism-related data from a rural county using an attention-based Long Short-Term Memory (Att-LSTM) model. The proposed study incorporated rural governance towards tourism development. The model improves rural tourist initiatives and bolsters rural rejuvenation, which makes essential contributions. The model examines data on customer satisfaction and finds that personal recommendations led to more promotion of visitors. According to the results, there are a few limitations to assessing and promoting rural tourism. These include the need for precise data and the model’s flexibility to handle various rural environments.

Expanding on tourism demand forecasting, Bi et al. [22] combined LSTM networks with Autoregressive (Autoreg) models to estimate tourism demand, using data from 77 sites in Beijing. The model increases forecast stability with better accuracy and less overfitting while capturing the spatial correlation among attractions. While the hybrid technique and new predictor combination are key contributions, there are certain constraints, such as potential concerns with data fidelity and the model’s adaptation to different scenarios.

Sulong et al. [23] used Bagged Classification and Regression Trees (BC-RT) to project the demand and economic efficiency of halal tourism demand in Malaysia. It analyses 338,233 tweets with emotion labels, 11 Google trend search terms, business-specific factors, and economic data covering 2009–2020. With an improved accuracy of 93.71% in demand prediction and 80.12% in financial performance prediction. Difficulty integrating different data sources and the possibility of bias are two limitations.

Turning to marketing strategies, Elgarhy et al. [24] applied the 7Ps of marketing services looks at how they impact customer happiness and loyalty using SEM. One significant contribution is its evidence for the partial mediation of customer happiness and loyalty in the relationship between business profitability and customer purchase intentions during travel. This information is beneficial for managers of tourism services. The most significant benefit is a thorough comprehension of the ability to use the 7Ps to improve connections with customers and increase profits. However, a possible drawback is the difficulty of precisely measuring and using all 7P components in varied and ever-changing tourist markets.

### 2.3 DL applications in tourism management and recommendations

Kyrylov et al. [25] examined the present situation and future possibilities of international tourism, paying special attention to Ukraine’s role in this industry. Significant results include a comprehensive analysis of world tourist indices, which place the United States, France, and Spain at the top. Research shows that tourism is a major driver of national economies, increasing GDP and bringing in much-needed foreign currency. Timely risk mitigation and decision-making rely on robust forecast models developed to anticipate future travel trends. While there is a need for improved risk mitigation techniques, the research also recognizes constraints to managing information support for economic entities in the tourism sector.

On the technological frontier, Wei [26] enhanced tourist attraction recommendation systems by extracting advanced image features, incorporating multimodal data fusion, and user portrait analysis using neural collaborative filtering in conjunction with deep convolutional neural networks (CNNs). Using a dataset including photos of popular tourist spots and information on user interactions allowed for an 89% success rate in picture recognition and an 85% success rate in recommendation-making. However, there are also certain restrictions, such as the difficulty of dealing with massive datasets and the need to adapt to a wide range of user preferences.

Siddik et al. [27] investigated the effect of artificial intelligence (AI) on the expansion of ecotourism using a mixed-method strategy that included Artificial Neural Network (ANN) analysis in addition to more conventional econometric models. From 2010 to 2022, the dataset encompassed the top ten tourist destinations worldwide, considering variables like GDP, FDI, inflation, and urbanization. While economic variables did have a role, the results demonstrated that AI was a major driver of tourism efficiency and sustainability. One restriction was the difficulty in applying the findings to various sites worldwide.

Srinivasan et al. [28] employed an adaptive attention mechanism and a transformer-based encoder to improve the sentiment classification of travel reviews. A diversified smart tourism dataset with user-generated information in multiple languages was used to train the model, which achieved an accuracy of 96.74%, higher than CNN-BiLSTM standards. Further optimization is needed for real-time applications due to limitations like computing complexity and the possibility of overfitting to domain-specific language.

The summary of existing research publications discussed in Table 1 that were evaluated include a wide range of approaches and applications that strive to improve tourist management and forecasting in various situations. Among these, you can find AI-driven marketing strategies for policy prediction after a pandemic, research using attention-based LSTM, models for forecasting tourism demand using hybrid LSTM-Autoreg, models for predicting demand in the halal tourism industry, models for understanding the impact of marketing on customer loyalty using SEM, and extensive analyses of tourism indices and economic impacts on a global scale. While these studies shed light on opportunities to enhance tourism plans and economic forecasts, they also shed light on obstacles, such as the need for more flexible models, better data integration, and better information management.

**Table 1:** Summary of Research Gap Analysis in Existing Works.

Reference	Algorithm	Contribution	Results	Limitation	How TourVaRNN Addresses Gaps
Polyakova et al [19]	Structural Equation Modeling (SEM)	Analyzed correlations between service quality and MTE dimensions.	The hedonic and local cultures were the strongest predictors of word-of-mouth behavior.	Downside due to event-specific and geographic focus.	TourVaRNN integrates VAE-RNN to model diverse segments and regional variations, enhancing generalizability.
Neshat et al. [20]	Deep Neural Network (DNN)	Policy-based tourism forecasting	Effective in policy suggestion and crisis handling	Limited adaptability to rapidly evolving situations	Uses recurrent structures to improve adaptability to dynamic tourism trends
Pan et al. [21]	Attention-based LSTM (Att-LSTM)	Enhances rural tourism governance	Boosts rural rejuvenation & tourist engagement	Requires highly precise tourism demand data.	TourVaRNN improves flexibility and can handle varying data precision across regions.
Bi et al. [22]	LSTM + Autoregressive Model	Tourism demand prediction with spatial correlation	More stable and accurate forecasting	Issues with data fidelity and generalization	Introduces variational autoencoders for better data representation and stability
Sulong et al. [23]	Bagged Classification & Regression Trees (BC-RT)	Demand and Economic Forecasting for Halal Tourism	93.71% accuracy in demand prediction	Bias risk and difficulty in integrating multiple data sources	Uses a variational framework to enhance data fusion and reduce bias
Elgarhy et al. [24]	Structural Equation Modeling (SEM)	Impact of 7Ps marketing on customer loyalty	Provides business insights for tourism managers	Lack of diverse market analysis	TourVaRNN integrates real-time analytics to adapt marketing strategies dynamically.
Kyrylov et al. [25]	Forecast Models	Global Tourism Trend Analysis	Identifies top tourism economies	Limited risk mitigation and data-driven decision-making	Enhances decision-making using predictive analytics for risk forecasting
Wei [26]	CNN + Neural Collaborative Filtering	Image-based attraction recommendations	89% accuracy in image recognition	Struggles with scalability and varied user preferences	Uses advanced embeddings to improve personalization and adaptability
Siddik et al. [27]	ANN + Econometric Models	AI’s impact on ecotourism	Demonstrates AI-driven tourism efficiency	Lacks applicability to diverse locations	TourVaRNN adapts to various tourism settings with regionalized training
Srinivasan et al. [28]	HTAM (Hybrid Transformer-Attention Model)	Integrates transformer and attention mechanisms for enhanced sentiment classification in smart tourism.	Achieved 96.74% accuracy, outperforming CNN-BiLSTM.	Prone to overfitting with domain-specific jargon; high computational cost.	TourVaRNN handles temporal patterns and uncertainty in tourist behavior, complementing sentiment insights with economic predictions.

## 3 Research methodology

The research aims to improve economic effect forecasts and give marketing strategy planners practical insights by using the proposed TourVaRNN model. Targeted marketing campaigns and policy decisions can be informed by in-depth analyses of tourist trends, employment effects, and expenditure patterns across various visitor types and locations, improving the local economy even further. To conduct an all-encompassing analysis of tourism data, the proposed Tourism Variational Recurrent Neural Network (TourVaRNN) system design incorporates variational autoencoders and recurrent neural networks. It includes layers to analyze inputs, encode hidden factors, anticipate requests, evaluate economic implications, and optimize to enable decisions based on information in tourism administration and marketing strategies. Analyze the direct and indirect effects of employment on tourism’s economic contribution. The exact methodology and appealing message of Tourism Economics highlight the crucial role of the visitor economy. Expenditure in marketing and infrastructure to assist tourist growth can be justified with an understanding of the larger contribution of tourism to the economy provided by economic impact data. In order to plan accommodation (places to stay), schedules and marketing to encourage longer stays, it is necessary to understand the duration of guests’ visits, and these indicators help with that. The goal of a visit can inform a marketing strategy’s personalization. While vacationers are searching for fun things to do, business travelers may be more interested in meeting spaces. As a prime instance, it is possible to divide marketing campaigns into separate ones for local and foreign visitors.

**Fig 1:**
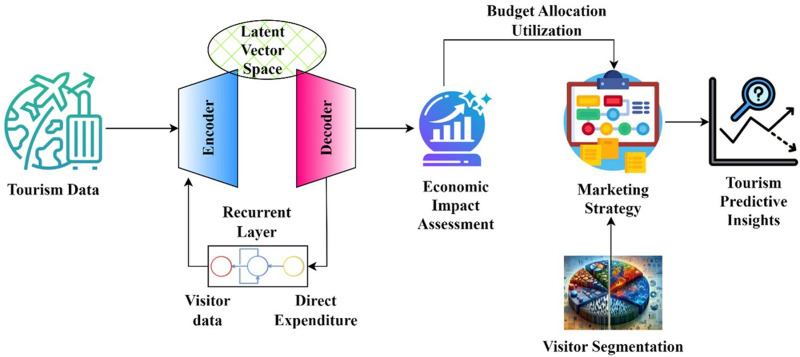
Schematic Representation of Proposed TourVaRNN Model.

This research idea depicted in Fig 1 adds to the knowledge of tourist analytics and economic prediction by introducing various novel ideas. The first thing you’ll notice about TourVaRNN is how it uses recurrent neural networks and variational autoencoders to pick up on uncertainty and delays in tourist data. Additionally, it breaks new ground in economic effect modeling by breaking down the contributions of visitors to employment and spending by age group. Thirdly, it offers an information-driven structure for developing marketing strategies by capitalizing on in-depth knowledge of consumer habits and economic trends. Fourthly, using latent variables, TourVaRNN improves visitor segmentation and provides detailed information on tourist preferences. Finally, the model suggests a new way to optimize the distribution of marketing funds by considering future economic effects and spending habits. Finally, TourVaRNN ‘s capacity to adjust to changing times suggests that it will be useful in the tourist industry for a long time to come, filling in gaps in current approaches and leading to improvements in tourism management and strategy. This study’s goal in doing visitor segmentation is to improve economic effect projections and marketing tactics by classifying tourists according to their demographics, interests, and behavioral tendencies.

### 3.1 Input tourism dataset description

The data employed for this research were sourced from the https://datamillnorth.org/download/visitor-economy-data/9fb31f0b-8c0c-4215-ac09-91f49adafbe2/Visitor%2520economy%2520data.csv [29], that encompasses a tourists visitor numbers, visitor days, average length of stay, expenditures, economic impact and employment figures across different years from 2017 to 2021 has been collected from the data source https://data.europa.eu/en/publications/datastories/high-value-datasets-tourism-eu [30] for performance analysis since it contains the necessary values for all attributes. This data includes domestic overnight visitors, day visitors, and overseas inbound visitors, with detailed information on spending patterns and international visit statistics including purpose of visit can be segmented by various regions. The dataset includes information on the volume of events, number of delegates, and gross value of business tourism. The hotel performance data includes hotel occupancy rates, and average room rates.

Table 2 discusses the features support the objectives of the study on marketing strategies and economic consequences by providing an all-encompassing view of tourist actions, revenue, and significant indicators pertaining to the tourist industry.

**Table 2:** Summary of Tourism Dataset.

Category	Details
Year of Analysis	2017-2021
Visitor Days	27.5M-32.1M days
Visitor Numbers	Maximum of 29M visitors/ year
Average Length of Stay	0.9-1.1 days
Visitors	Domestic Overnight Visitors, Day Visitors, Overseas Inbound Visitors
Economic Impact	Direct Expenditure (£M)
Employment Impact	Direct, Indirect (jobs)
International Visits	North America, Europe, Other Countries and Total world
	Average Nights Stayed, Average Spend Per Visit, Average Spend Per Day
Demographics (Age groups)	(0-15), (16-24), (25-34), (35-44), (45-54), (55-64), >65
Purpose of Visit	Holiday, Part of Inclusive Tour, Business, Visiting Friends & Relatives (VFR), study, Others
Business Tourism Value (£)	(£) 409.5M- (£)577.7M

#### Data cleaning and handling outliers.

Data cleaning includes handling missing values and outlier detection, where tourism data often contains missing values due to incomplete surveys, reporting errors, or seasonal variations. The numerical data imputation using mean/median considers from


$$X_{missing}=\frac{\sum_{i=1}^{N}X_{i}}{N}$$ (1a)

The mode imputation for visitor categories replaces missing purpose of visit values with the most frequent category. Outliers in tourism datasets can arise due to data entry errors, extreme events like pandemic impact and for detecting these anomalies, apply z-score method. Preventing anomalies form misclassifying visitor groups based on spending behavior and stay duration discussed in Eqns. (1a) and (1b)


$$Z=\frac{X-\mu }{\sigma }$$ (1b)

If Z>3, the data point is considered an outlier, and is either removed or replaced using interpolation.


$$IQR=Q_{3}-Q_{1};Q_{1}=25^{th}percentile\left(l\right),Q_{3}=75^{th}percentile\left(u\right)$$

Where


$$l=Q_{1}-1.5\times IQR;u=Q_{3}+1.5\times IQR$$

The data points outside this range are flagged as outliers. Thus, sudden seasonal peaks or falls in hotel occupancy and visitor numbers could mislead model predictions. Also, the international visit trends like political factors, exchange rate fluctuations might cause extreme variations.

### 3.2 Variational encoder process

From the provided dataset the variables of interest given for visitor numbers termed as *V* can be formulated as


$$V=\left[v_{1},v_{2},v_{3},\ldots ,v_{t}\right]$$ (2)

where $v_{t}$ in Eq. (2), represents the number of visitors at time period *t* indicating in terms of years. An average length of stay can be represented as $L=\left[l_{1},l_{2},l_{3},\ldots ,l_{t}\right]$ where $l_{t}$ denotes the average length of stay at time *t* indicating in terms of particular years. Likewise, economic impact $,E_{I}=\left[e_{i1},e_{i2},..,e_{it}\right]$ varied with respect to specific year with in a given range, and for different age group *i*. For encoder process the variational layer model’s latent variable $z_{t}$ indicates visitor preferences, spending habits, and economic impact drivers at time *t* is used to comprehend and forecast tourist economic results, these unseen elements are vital and can be defined in Eq. (3a & 3b).


$$q\left(z_{t}\text{|}x_{t}\right)=N\left(z_{t}|\mu_{t},\sigma_{t}^{2}\right)$$ (3a)

$$z_{t}\sim p(z_{t}|z_{t-1,}h_{t-1})$$ (3b)

The variational distribution $q\left(z_{t}\text{|}x_{t}\right)$ captures the uncertainty in the $z_{t}$ in Eq. (3) gives the observed data $x_{t}$ like visitor demographics, visitor days and other attributes that impacts the economic assessment.

### 3.3 Latent vector space representation

For the tourist data, the latent vector component models include latent variables, that stand for undiscovered trends and unidentified factors. It uses variational methodologies to capture complicated correlations and variances in elements impacting economic impacts and tourist behaviors, such as visitor preferences and spending patterns. In order to analyze demographic information and purpose of visit data for tourism, deep learning’s hidden layers store abstract traits and patterns, which allows for precise demand forecasting and strategic decision-making.

The latent variables are expressed as Latent Variable 1 (Age Group): To create age-specific marketing campaigns, it is necessary to capture visitor age segments (e.g., children, adults, seniors). The second latent variable, “Spending Behavior,” shows how people spend their money, differentiating between those on a tight budget and those able to splurge. Third latent variable, “travel purpose,” sorts visitors into three categories: vacation, business, and family. This allows for more personalized advertising. Tracks patterns of recurrent visits to facilitate the creation of loyalty programs; this is latent variable 4 (Frequency of Visits). The process of segmentation involves visualizing groups of people in order to conduct targeted marketing, such as high-spending leisure travelers. Campaign optimization: Maximizes engagement and conversions by coordinating marketing campaigns with visitor behaviors.

**Fig 2:**
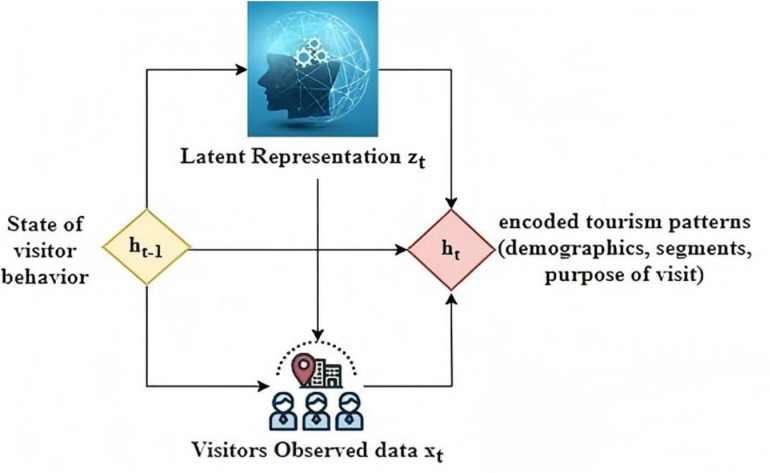
Learning Tourism Patterns from Hidden State Representation.

Fig 2 incorporates the variational autoencoder at each time step of the recurrent layer and the non-determinism presents in the hidden state $h_{t}$ helps to capture the variability of the input state of visitor sequence and generates robust output distribution of the identified tourism patterns.

### 3.4 Recurrent layer for analyzing visitor behavior

Using Recurrent Neural Networks (RNNs), the TourVaRNN model’s recurrent layer records the interdependencies between visitor behavior and economic effect data over time. This layer’s ability to keep a secret state including past data is what gives the model its trend understanding and prediction capabilities. The accuracy of future economic effect and visitor behavior estimates is enhanced by the recurrent layer, which leverages historical trends. In the tourist sector, this competence aids in more informed strategic planning and decision-making. The following mathematical formulation serves as a contextual information that influences the computation of the current hidden state $h_{t}$ and subsequent predictions or decisions in the model.


$$h_{t}\left(V\right)=f\left(h_{t-1},z_{t},x_{t}\right)$$ (4)

This Eq. (4), defines the function $h_{t}\left(V\right)$of all previous inputs and ensures that both the variability of the input sequence and temporal dependence between $z_{t}$ up to the current time step across the distinct time period is captured. The inner function of $x_{t}$ acts as a feature extractor and captures the $l_{t}$ and $v_{t}$. The term $h_{t-1}$ represents the state of visitor behavior and trends up to the previous point in time, aiding in understanding and predicting future patterns based on past data.

**Fig 3:**
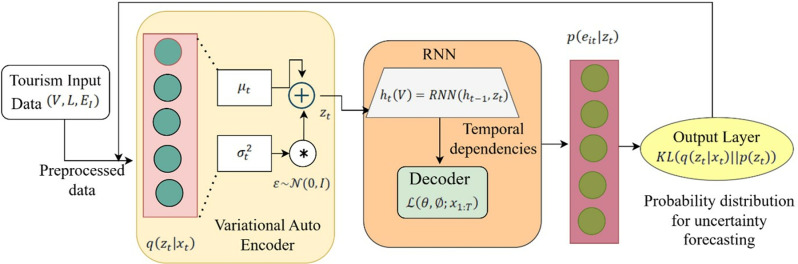
Integration of VAE and RNN Architecture.

By combining VAE with RNN, the architecture shown in Fig 3 known as the TourVaRN models the temporal interdependence and uncertainty in tourist data forecasting. After the autoencoder $q\left(z_{t}\text{|}x_{t}\right)$ from Eq. (2) converts the input sequences $V,L,E_{I}$ to a latent space, stochastic relationships are captured by variational inference. The recurrent decoder uses learnt latent representations to recover future states. This design optimizes uncertainty quantification and temporal dependencies simultaneously, which guarantees strong forecasting. Include assessment feedback in the data preprocessing block, suggesting that learning from model performance could lead to additional preprocessing step refining.

### 3.5 Variational decoder process

For decoder process the reconstruction layer can be used to predict economic impact $e_{it}$ based on $z_{t}$ is derived using an Eq. (5). In charge of using the model’s learned encoded latent variables and temporal dependencies to reconstruct economic impacts and make predictions about visitor actions. Decisions can be made with more knowledge on the impact of changing visitor demographics and habits on economic outcomes.


$$p\left(e_{it}|z_{t}\right)=N\left(e_{it}|{\hat{e}}_{it},\varepsilon \right)$$ (5)

The term $p\left(e_{it}|z_{t}\right)$ predicts economic impacts based on learned latent representations $z_{t}$, offering insights into how various other factors like visitor behaviors and spending patterns influence economic outcomes.

### 3.6 Economic impact assessment

The direct spending and the effect of tourism on jobs are examined in this section. In doing so, it hopes to provide stakeholders with vital criteria for gauging the sector’s whole economic health and possible growth areas by quantifying the economic benefits of tourism activities. By using variational lower bound, the elbow variational lower bound *L* derived in Eq. (6) optimizes the model parameters *θ* and  ∅  of the inference network to accurately predict economic impacts while ensuring that the latent variables capture meaningful patterns in the data.


$$L\left(\theta ,\emptyset ;x_{1:T}\right)=-E_{q\left(z_{t}\text{|}\mu_{t}\right)}\left[\log p\left(e_{it}|z_{t}\right)\right]+KL\left(q\left(z_{t}|x_{t}\right)||p\left(z_{t}\right)\right)$$ (6)

This method optimizes the model by balancing reconstruction accuracy with the regularization term KL divergence. This is crucial for robustly modeling tourism economics, ensuring that the model learns ${\text{z}}_{\text{t}}$ aligns with the observed ${\text{x}}_{\text{t}}$ data. The variable ${\text{μ}}_{\text{t}}$ defines the mean vector and ${\text{σ}}_{\text{t}}^{2}$ defines the variance -covariance matrix, indicating where ${\text{z}}_{\text{t}}$ is likely to lie in the latent space. The conditional probability distribution $\text{p}\left({\text{e}}_{\text{it}}|{\text{z}}_{\text{t}}\right)$ defines the ${\text{e}}_{\text{it}}$ given ${\text{z}}_{\text{t}}$ assumes Gaussian distribution parameterized by ${\hat{e}}_{it}$ as mean prediction and ε as variance.


$$KL\left(q\left(z_{t}|x_{t}\right)||p\left(z_{t}\right)\right)=\frac{1}{2}\overset{N}{\underset{i=1}{\sum }}\left(1+log\left({\left(\sigma_{t}^{2}\right)}_{i}\right)-{\left(\mu_{t}\right)}_{i}^{2}-{\left(\sigma_{t}^{2}\right)}_{i}\right)$$ (7)

Where in Eq. (7), $KL\left(q\left(z_{t}|x_{t}\right)||p\left(z_{t}\right)\right)$ defines the approximate posterior $q\left(z_{t}|x_{t}\right)$ is close to the prior $p\left(z_{t}\right)$ in the latent space and it is computed element-wise across the dimensions of $z_{t}$ and also the variable *N* defines the dimensionality of $z_{t}$.


$$E_{I}=\left(\frac{s_{i}\times P_{i}\times W_{i}}{tot\left(E_{I}\right)}\right)\times 100$$ (8)

Utilizing the economic impact $E_{I}$ calculated from Eq. (8) helps for each group *i* is calculated by integrating predicted total spending $s_{i}$ from analyzed visitor data from encoder process, the proportion of the population $P_{i}$ represented by that age group, and a weighted factor $W_{i}$ that signifies the economic influence of each group. By adjusting the weights based on empirical data the assessment reflects varying spending behaviors from direct Expenditure.

### 3.7 Visitor segmentation

Using the acquired latent variables, it divides visitors into segments according to their demographics and how they engage. In order to increase customer happiness and financial returns, it is helpful to segment visitors based on their preferences. This allows for more targeted marketing and better services. From Eq. (9), latent variables $z_{t}$ to segment visitors based on spending behavior, demographics, and purpose of visit like identifying the travel motivations.


$$segment\left(t\right)=argmax_{k}\left(p\left(segment=k|z_{t}\right)\right)$$ (9)

$$p\left(segment_{k}\text{|}z_{t},a_{g}\right)=\frac{\text{exp}\left(W_{k}^{T}z_{t}+b_{k}+V_{k}^{T}a_{g}\right)}{\sum_{j=1}^{K}\text{exp}\left(W_{j}^{T}z_{t}+b_{j}+V_{j}^{T}a_{g}\right)}$$ (10)

In Eq. (10), the mathematical formulation segments visitors into *K* different categories *k* based on latent variables $z_{t}$ and age group features termed as $a_{g}$. The other indicator variables like $W_{k},b_{k},andV_{k}$ are learnable parameters for each segment, determining the probability of a visitor belonging to segment *k* for evaluating marketing strategy. Apply these segments to forecast spending patterns ${\hat{s}}_{t+1}\left(k\right)$ for targeted marketing. The spending data comprises Expenditure per visit, average spend per day to forecast future spending. Incorporate these predictions into spending patterns $\underset{k}{\sum }{\hat{s}}_{t+1}\left(k\right)$ for budget allocation. For allocatingthe marketing budget $B_{t}$ at time *t* derive the Eq. (11) of the form.


$$B_{t}=\alpha {\hat{e}}_{t+1}+\beta \underset{k}{\sum }{\hat{s}}_{t+1}\left(k\right)$$ (11)

The parameters *α* and *β* indicates the weights that can be adjusted based on strategic priorities and model predictions. The term *α* indicates the weight reflecting the importance of predicted economic impact ${\hat{e}}_{t+1}$, similarly *β* represents the weight reflecting the importance of spending patterns ${\hat{s}}_{t+1}\left(k\right)$ of segment *k* at time t+1, would prioritize revenue optimization from specific visitor segments.

### 3.8 Pseudocode for visitor segmentation

**Table:** 

**function** VisitorSegmentation ($z_{t},a_{g}$): Init parameters ($K,W_{k},b_{k},V_{k}$) ** for** k=1toK **do** # Segment probability calculation compute $\text{exp}\left(W_{k}^{T}z_{t}+b_{k}+V_{k}^{T}a_{g}\right)$ compute $\overset{K}{\underset{j=1}{\sum }}\text{exp}\left(W_{j}^{T}z_{t}+b_{j}+V_{j}^{T}a_{g}\right)$ $segment\left(t\right)$ = $argmax_{k}p\left(segment_{k}\text{|}z_{t},a_{g}\right)$ ** end for** **return** $segment\left(t\right)$ **function** ForecastSpendingPatterns $\left(segment,t\right)$: ** for** k=1toK **do** # Forecast spending for each segment ${\hat{s}}_{t+1}\left(k\right)$ = PredictSpending ($segment_{k}$, t+1) **end for** **return** $\underset{k}{\sum }{\hat{s}}_{t+1}\left(k\right)$ **function** AllocateMarketingBudget(${\hat{e}}_{t+1}$, ${\hat{s}}_{t+1}\left(k\right)$): init (α,β) compute $B_{t}$ **return** $B_{t}$ **function** OptimizeBudgetAllocation(*K*, totalbudget): init Rev=0 evaluate $B_{k}=0forkinrange\left(K\right)$ **While** $B_{k}\leq totalbudget$ **do** **for** k=1toK **do** **if** $B_{k}\geq MinBudget_{k}$ **then** $ROI_{k}=calcualteROI\left(k,B_{k}\right)$ $Rev+=\alpha_{k}\cdot B_{k}\cdot ROI_{k}$ **else** $B_{k}>totalbudget$ **end for** **return** $Rev,B_{k}$ # Main segmentation and budget allocation process $z_{t}=$ GetLatentVariables(visitor_data) $a_{g}$ = GetAgeGroupFeatures(visitor_data) segment = VisitorSegmentation($z_{t}$, $a_{g}$) ${\hat{s}}_{t+1}$= ForecastSpendingPatterns (segment, current_time) ${\hat{e}}_{t+1}$= ForecastEconomicImpact($z_{t}$) $B_{t}$= AllocateMarketingBudget(${\hat{e}}_{t+1}$, ${\hat{s}}_{t+1}$) totalbudget = $B_{t}$ **return** $tot\left(E_{I}\right)$

The provided pseudocode outlines a structure for the tourist industry’s visitor segmentation and marketing budget allocation. It separates visitors by latent characteristics and age group, forecasts spending habits, and optimizes expenditures on marketing. This technique targets marketing strategies and optimizes resource allocation, improving tourism marketing’s economic impact and efficacy. The VisitorSegmentation function assigns visitors to the most likely segment using latent variables $z_{t}$ and age group attributes $a_{g}$. ForecastSpendingPatterns anticipates segment spending trends and totals them. OptimizeBudgetAllocation distributes the budget across segments to optimize revenue. The core procedure uses visitor data to identify hidden characteristics and demographic features, segment visitors, forecast expenditure and economic impact, distribute the marketing budget, and return the total predicted economic impact $tot\left(E_{I}\right)$.

### 3.9 Marketing strategy formulation

Marketing strategy formulation optimizes marketing budget allocation and maximizes the revenue across various visitor segments and creates strategic campaigns. Optimizing marketing efforts and improving visitor experiences are the goals of this strategy formulation, that draws on data from demand forecasting and visitor segmentation.

### 3.10 Budget allocation utilization

The Budget Allocation Utilization (BAU) influenced by market potential, historical performance, and strategic priorities.


$$MaximizeRev=\overset{K}{\underset{k=1}{\sum }}\alpha_{k}\cdot B_{k}\cdot ROI_{k}$$ (12)

subject to the constraints:


$$\overset{K}{\underset{k=1}{\sum }}\begin{array}{c} B_{k}\leq totalbudget\\ B_{k}\geq MinBudget_{k}\forall k \end{array}$$

The term $\alpha_{k}$ represents weight for segment *k* based on its strategic importance and $B_{k}$ indicates budget allocated to segment *k* followed by $ROI_{k}$ represents return on investment for segment *k*. The goal is to maximize total revenue by allocating the marketing spend optimally across different categories. The given constraints make sure there is enough money in the budget for everything and that it doesn’t go overboard. More efficient use of marketing resources can increase income, which can be achieved through optimizing budget allocation.

From Table 3 demonstrates the data from time period 2017–2021 provide insights into international visitors, that could inform both economic analysis and marketing strategies for different regions. Tourism officials can maximize revenue through targeted marketing and personalized service offers by analyzing the spending patterns ${\hat{s}}_{t+1}\left(k\right)$ and stay durations of visitors across various regions. Developing regional advertising strategies to entice North American tourists with large spending power and international visitors with extended stays $l_{t}$. Creating discounted long-term packages and shorter-stay options for tourists from Europe and other continents. Improving the tourist experience according to local tastes and spending habits to increase happiness and encourage return visits.

**Table 3:** International Visitor Analysis for Marketing in Different Regions.

Region	Avg. Nights Stayed$l_{t}$	Avg. Spend per visit (£)	Avg. Spend per Day (£)
North America	7	617	83
Europe	4	244	64
Other Countries	13	910	68
Total World (Finland, Romania, Poland, Spain, Germany, Greece)	6	398	69

## 4 Results and discussion

For comparison study the existing models such as DNN [20], Att-LSTM [21], and BC-RT [23] are contrasted with the proposed TourVaRNN in terms of performance metrics such as economic impact assessment, visitor segmentation efficiency, inference time analysis, and budget allocation utilization for effective economic and marketing strategy analysis in the tourism industry.

TourVaRNN surpasses DNN in performance due to its superior uncertainty modeling using VAEs, less need for human feature engineering, and improved predictive accuracy, as shown in Table 4. It provides shorter inference times for real-time forecasting and efficiently captures long-term patterns compared to Att-LSTM. Compared to BC-RT, TourVaRNN is superior in adaptability to visitor behaviors, output reliability, and probabilistic outputs.

**Table 4:** Comparison of Models: Limitations Vs. TourVaRNN Advantages.

Model	Limitations	TourVaRNN Advantages
**Deep Neural Network (DNN)**	• Overfitting risk with limited data.	• VAEs quantify uncertainty for reliable forecasting.
	• Requires extensive feature engineering. • Lacks uncertainty quantification.	• Requires less manual feature engineering and enhance predictive accuracy.
**Attention-Based Long Short-Term Memory (Att-LSTM)**	• Computationally expensive due to attention mechanisms.	• Efficiently captures long-term temporal patterns with faster inference times for real-time prediction
	• Struggles with long-term dependencies without proper tuning. • Requires large, high•quality datasets.	• Optimized inference speed for real•time predictions.

**Bagged Classification and Regression Trees (BC-RT)**	• Susceptible to bias from training data and lacks accuracy	• More flexible and adaptive in learning complex visitor behaviors and improves forecast accuracy
	• Rigid structure limits adaptation to nonlinear relationships. • Lacks uncertainty modeling.	• Provides probabilistic outputs for better decision-making.

For this comparison analysis, two variants are selected; one is visitors’ demographics from [29] based on different age groups (0–15), (16–24), (25–34), (35–44), (45–54), (55–64), >65. The second variant is based on tourism data analysis from [30] from 2017 to 2021.

Table 5 presents the key hyperparameters for the TourVaRNN model. It uses a learning rate of 0.0001, 128 hidden neurons, and two layers with a batch size of 64 for training over 100 epochs. The model employs 30-time steps, early stopping patience as 10, and a latent space of 64 for effective sequence learning and economic impact prediction.

**Table 5:** Hyperparameter Parameter Setting.

Parameter	Range with its Description
**Learning Rate**	0.0001- Rate of update for model weights.
**Hidden Layer Size**	128 - Number of neurons in each hidden layer.
**Number of Layers**	2 (VAE+RNN) - Total recurrent and variational layers in the model.
**Batch Size**	64 - Number of samples per gradient update.
**Training Iterations**	100 - Total epochs for training the model.
**Loss Function**	Mean Squared Error (MSE) - Metric for evaluating model performance.
**Sequence Length**	30 - Number of time steps taken as input to the RNN.
**Early Stopping Patience**	10 - Number of epochs without improvement before stopping training.
**Latent Space Dimension**	64 - Dimensionality of latent variables in the variational autoencoder.
$W_{k},b_{k},andV_{k}$	Learnable parameters

### 4.1 Tourism predictive insights

Direct Expenditure (£M) is the accumulation of all the money tourists spend in a particular region. Everything directly linked to the tourists’ spending falls under this category, including Accommodation: The amount of money spent on various forms of housing, including hotels, hostels, bed & breakfasts, and more. Expenditures on food and drink at multiple establishments such as restaurants, cafes, and bars. Transportation: Expenses related to getting around town include taking the bus, a cab, renting a car, and gas—expenditures on amusements, museums, tours, performances, and other entertainment. Merchandise tourists buy may include apparel, trinkets, and other mementoes. Miscellaneous Expenses: Tips, medical services, and personal care are examples of visitor expenditures that cannot be categorized into the above categories.

As tourists’ spending grows, so does the overall economic contribution because of the positive link between direct Expenditure and economic impact. The correlation analyzed below suggests that fluctuations in direct Expenditure can serve as a predictive factor for economic impact, reinforcing the importance of tourism as a key economic driver.


$$E_{I}=\gamma_{0}+\gamma_{1}{\hat{s}}_{t+1}\left(k\right)+\varepsilon$$ (13)

Where $\gamma_{0}$ in Eq. (13) represents the marginal economic impact per unit increase in tourist spending, A positive $\gamma_{1}$ confirms a direct proportionality, meaning increased expenditure leads to a higher economic impact. where $\gamma_{1}>0$ confirms that higher visitor spending leads to a directly proportional increase in economic impact. The statistical significance is typically validated using regression analysis, where a high R^2^ value and a p-value < 0.05 indicate a strong and meaningful relationship.

**Fig 4:**
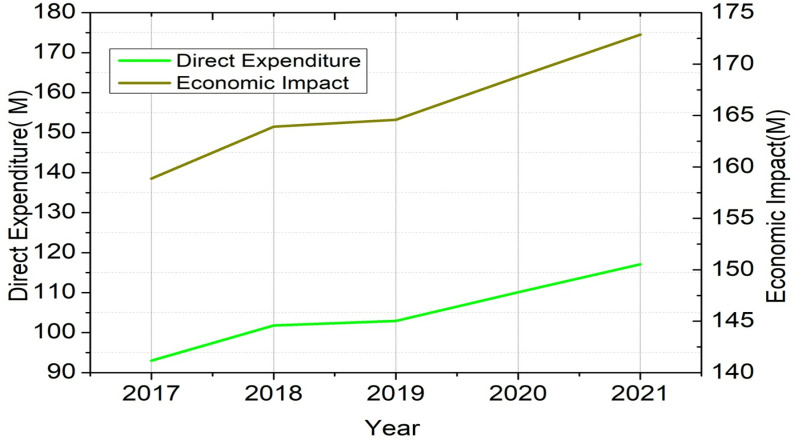
Direct Expenditure Vs. Economic Impact (2017-2021).

The graphical illustration provided in Fig 4 shows that the direct spending by visitors correlates with the overall economic impact over the years. As direct expenditure increases, there will be a corresponding rise in the economic impact, suggesting a strong positive relationship between these two variables. The left y-axis indicates the direct Expenditure (£M) evaluated for millions of pounds sterling, and the right y-axis defines the economic impact in terms of (£M). Demand forecasting helps future visitor numbers V, and visitor days can be given as ${\hat{v}}_{t+1}$ can be evaluated using the proposed TourVaRNN to predict future demand.

#### i) Economic impact assessment.

For evaluating economic impact assessment among different algorithms, the proposed TourVaRNN outperforms DNN, Att-LSTM, and BC-RT in economic impact assessments for most age groups. This shows that TourVaRNN’s capacity to capture complex patterns using variational inference blocks for analyzing spending patterns $s_{i}$ Concerning consequences across many age groups, they lead to more precise forecasts.


$$E_{I}=\left(\frac{s_{i}\times P_{i}\times W_{i}}{tot\left(E_{I}\right)}\right)\times 100$$ (14)

On the other hand, DNN, Att-LSTM, and BC-RT all show different levels of economic impact $E_{I}$ derived in Eq. (14) depends on age group, which shows how well they model and predict economic dynamics within specific demographics varied on different $W_{i}$. Policymakers and tourist managers rely heavily on these findings for long-term planning. To maximize the economic benefits of tourism, officials need to know which age groups have the most significant influence on the industry’s bottom line. Only then can they modify marketing campaigns, distribute budget funds wisely, and plan for infrastructure upgrades accordingly based on these $s_{i}\times P_{i}\times W_{i}$ evaluation factors for economic analysis. The results also show the importance of using advanced DL techniques with the combined effort of the variational auto encoder and recurrent neural network as TourVaRNN to improve economic effect evaluations and back decisions in tourism and economic planning with data.

Based on the computed percentage improvement provided in Table 6, which comprises roughly 7.63%, TourVaRNN has a higher economic impact assessment across the age group of 0–15 than DNN. This method gives a holistic picture of how various age groups impact tourism’s bottom line. Stakeholders can allocate budgets effectively and enhance the economic advantages received from tourism operations with the help of this methodology, which evaluates the economic impact of different age groups and guides strategic decisions in the management of tourism and marketing.

**Table 6:** Economic Impact Assessment by Age Group Comparison.

Age Group	DNN	Att-LSTM	BC-RT	TourVaRNN
(0-15)	11.8	10.5	12.0	12.7
(16-24)	14.5	13.2	15.1	15.6
(25-34)	17.9	18.3	17.5	19.4
(35-44)	19.6	20.1	20.0	21.9
(45-54)	18.9	19.5	18.7	20.7
(55-64)	16.7	17.0	16.8	18.5
>65	15.8	15.2	16.0	17.3

This research applies analysis of variance (ANOVA) to determine if there are statistically significant variations in the mean $E_{I}$ evaluations according to $a_{g}$.

***Key Steps in ANOVA Implementation.* Formulation of Hypothesis**:

Null Hypothesis ($H_{0}$)**:** $E_{I}$ means across all $a_{g}$ are equal, indicating no significant influence of age on economic contribution.

Alternate Hypothesis ($H_{1}$): At least one $a_{g}$ has a significantly different mean economic impact, suggesting that age group affects tourism spending behavior $s_{i}$.

The F-statistic is calculated by comparing variance between and within age groups, and if a high F-value with a p-value<0.05 indicates the difference in $E_{I}$ across age segments.

There are noticeable disparities in the economic impact shown in Table 7, with the 25–44 age group making the most significant contribution, suggesting that they spend more on tourism. The preferences of active travel groups (16–64) exhibit moderate to high diversity.

**Table 7:** ANOVA Validation for Economic Impact Across Age Groups.

Age Group	Mean Economic Impact	Variance (Within Group)	Variance (Between Groups)	Possible Reasons for Economic Impact Differences
**0-15**	12.7	Low	High	Lower spending power due to financial dependence on guardians.
**16-24**	15.6	Moderate	High	Young adults travel for education, adventure, and social activities.
**25-34**	19.4	High	High	Peak earning phase, higher disposable income, frequent travelers.
**35-44**	21.9	High	High	Family-oriented travel, business trips, and higher spending on accommodations.
**45-54**	20.7	Moderate	Moderate	Work-life balance, moderate disposable income, and steady tourism participation.
**55-64**	18.5	Moderate	Moderate	Retirement planning and leisure trips but budget-conscious spending.
**>65**	17.3	Low	Moderate	Fixed incomes lead to lower travel spending and a preference for relaxed tourism.

#### ii) Visitor segmentation efficiency analysis.

Through behavioural and preference-based tourist segmentation, TourVaRNN facilitates the development of targeted marketing campaigns. Engagement and conversion rates can be enhanced with this tailored strategy. Proactive marketing that meets expected demand, making the most efficient use of resources while increasing impact, is made possible by the model’s predictions of future visitor behaviors and spending habits.

**Fig 5:**
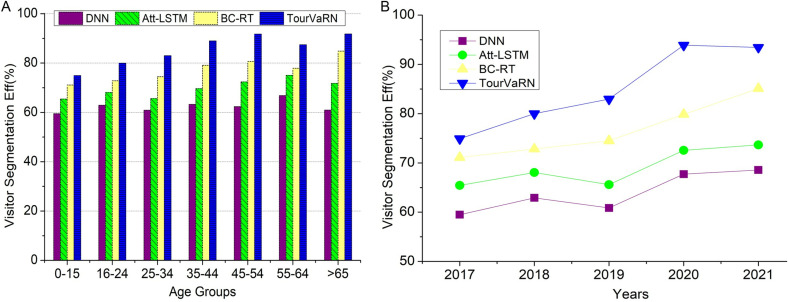
(a). The Efficiency of Visitor Segmentation Over a Different Age Population. (b). Efficiency of Visitor Segmentation Over a Year.

An essential measure for assessing the efficacy of segmentation tactics in marketing and tourism is visitor segmentation efficiency, depicted in Figs 5(a) and 5(b). Companies can improve their tactics to increase consumer engagement and economic effect by measuring the accuracy with which segments represent visitor diversity and forecast behaviors. The proposed TourVaRNN patterns and places to improve your segmentation techniques by plotting the efficacy of visitor segmentation over time or across many different categories, such as age groups and across different years). If the segmentation approach can capture all the variations and nuances throughout visitors, it allows for more targeted marketing activities and resource allocations, resulting in higher efficiency.

The TourVaRNN model provides visitor spending forecasts across segments, exhibiting varying uncertainty levels in Table 8. Leisure and family vacation segments demonstrate good reliability with narrower confidence ranges. There is a moderate level of dependability in business travel and group tours because of the higher variance. With less variation and a narrower confidence range, couples’ getaways are the least reliable.

**Table 8:** Confidence Intervals and Variance Analysis for TourVaRNN Predictions.

Segment	Prediction	Mean Prediction Value	Confidence Interval (95%)	Variance	Reliability Indicator
Segment 1 (Leisure Travel)	Visitor Spending	$800	750–850	100	High
Segment 2 (Business Travel)	Visitor Spending	$1200	1150–1250	150	Moderate
Segment 3 (Family Vacations)	Visitor Spending	$950	900–1000	200	High
Segment 4 (Couples’ Getaways)	Visitor Spending	$700	650–750	80	Low
Segment 5 (Group Tours)	Visitor Spending	$600	550–650	250	Moderate

Table 9 presents four types of visitors, each with its traits, spending habits, and validation data from real-world tourism marketing campaigns. The marketing campaign outcomes match each segment’s profile, including average stay, spending habits, and preferences. The segmentation model is validated and found to be practically relevant when segment behaviors align with marketing outcomes like return on investment (ROI) from promotions or program conversions.

**Table 9:** Visitor Segmentation Validation Accuracy.

Segment	Characteristics	Spending Behaviors	Validation (Marketing Outcome Comparison)
**Segment 1: Luxury Travelers**	High-income individuals, average, stay 10 nights	Average spend £950 per visit, prefer premium experiences	Matches luxury campaign results, showing increased ROI from high-end offers
**Segment 2: Budget Travelers**	Cost-conscious tourists, average stay 5 nights	Average spend £250 per visit, prefer economical packages	Aligns with budget campaign results, showing strong conversions from promotions
**Segment 3: Business Travelers**	Frequent short-stay visitors, typically for work trips	Average spend £300 per visit, high daily spend on accommodations and transport	Correlates with business incentive program outcomes
**Segment 4: Family Travelers**	Group travellers, average stay 7 nights	Average spend £600 per visit, seek family-friendly services	Consistent with outcomes of family-oriented package promotions

#### iii) Inference time calculation.

TourVaRNN fared better than similar models such as DNN, Att-LSTM, and BC-RT in conducting economic effect assessments across various age groups. It proved more efficient in distinguishing between different types of visitors, which made targeted marketing and resource allocation possible. Additionally, TourVaRNN demonstrated the quickest inference times, which facilitated the implementation of real-time decision-making in the tourism management sector. The model’s full research informs strategic marketing campaigns and policy decisions regarding tourist trends, employment effects, and expenditure patterns. This analysis can strengthen local economies while also providing valuable insights.

**Fig 6:**
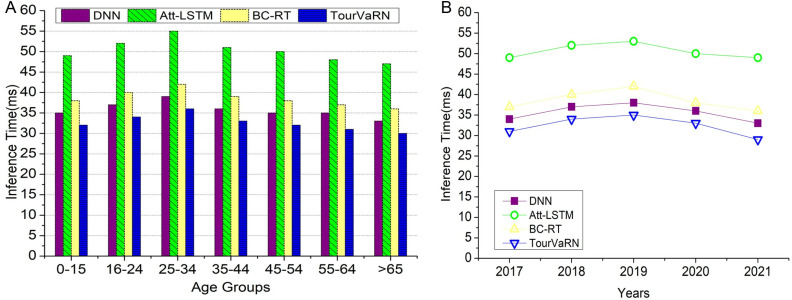
(a). Inference Time Analysis on Distinct Age Groups. (b). Inference Time Analysis Across Years.

Regardless of age in Fig 6(a) and year in Fig 6(b), the TourVaRN model consistently displays the quickest inference times. The fact that it outperforms simpler models despite its complexity is a result of its streamlined architecture and efficient use of variational inference. Except for TourVaRNN, which keeps its maximum time of 40 ms, this is the highest limit of the inference time, which is set to 60 ms for all models. TourVaRNN ‘s reduced inference times indicate it can handle tourism data faster, allowing for quicker decision-making in marketing and management strategies and real-time applications. The proposed model’s capacity to simulate temporal changes and integration of age group data makes it ideal for studying tourism patterns from 2017 to 2021. It may show changing trends in visitor segments based on age. From 2017 to 2021, inference times generally decreased, which could be due to hardware advancements or model optimizations.

#### iv) Budget allocation utilization.

**Fig 7:**
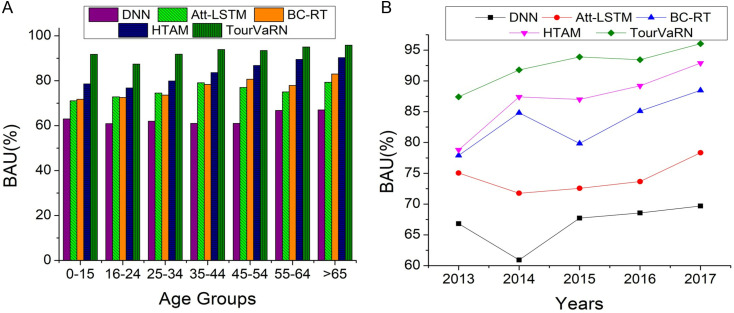
(a). BAU Comparison on Different Age Groups. (b). BAU Comparison on Varying Years.

Using this BAU calculation illustrated in Figs 7(a) and 7(b), the efficiency of each algorithm distributes funds among different demographic subsets and time intervals like years. For each age group and year, analyze each algorithm, including proposed TourVaRNN, compared to existing DNN, Att-LSTM, BC-RT, and HTAM models to forecast the number of visitors or economic effect. Consider the unique traits with the constraints and check $B_{k}\leq totalbudget$ and tendencies detected by each algorithm when allocating funds according to these forecasts. The continuous analysis of the marketing budget varies on $B_{k}\geq MinBudget_{k}\forall k$ and how much revenue can be generated throughthis marketing strategy formulation $\overset{K}{\underset{k=1}{\sum }}\alpha_{k}\cdot B_{k}\cdot ROI_{k}$ and analyze actual $B_{k}$ spent on each agegroup and year. Evaluate the algorithms’ BAU values across various demographic groupings and periods to determine which generates higher utilization rates. Determine the efficacy of each algorithm by examining patterns and trends in BAU. Identifies when and where algorithms show a higher BAU, aiding marketing strategy optimization. converts forecasts into efficient distributions of funds.

**Case Study: 1 High-Spending Behavior in Certain Age Groups**

Travellers aged 30–45 were shown to exhibit high-spending tendencies in a regional tourism dataset analyzed with TourVaRNN. The emergence of premium tourist products might be attributed to the fact that this age group spends 35% more on leisure activities than younger travelers (Table 10).

**Table 10:** Segment-wise Spending Analysis.

Age Group	Average Expenditure ($)	Percentage Difference
18-29	100	–
30-45	135	**35%**
46-60	120	**20%**
60+	110	**10%**

**Fig 8:**
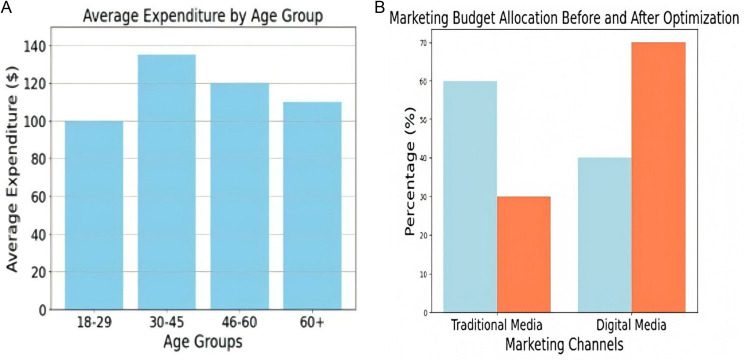
(a) Avg Expenditure Vs. Age Groups (b) Fig. Dynamic Budget Allocation.

Figs 8(a) and 8(b) visualize spending behavior among age groups and marketing budget allocation before and after optimization. The targeted campaign promotes premium experiences such as exclusive tours and luxury accommodations for the 30–45 age group in Table 11.

**Table 11:** Marketing Campaign Analysis.

Metric	Before Campaign	After Campaign	Increase
Marketing Investment ($)	0	1,50,000	–
Revenue ($)	0	6,00,000	400% ROI

**Case Study 2: Dynamic Budget Allocation**

TourVaRNN redirected resources to better-performing platforms after comparing the effectiveness of digital and conventional marketing channels.

Table 12 provides shifted marketing spend to social media platforms, demonstrating higher engagement among younger demographics.

**Table 12:** Dynamic Budget Allocation Analysis (Pre-Post Optimization).

Marketing Channel	Pre-Optimization (%)	Post-Optimization (%)
Traditional Media	60%	30%
Digital Media	40%	70%

## 5 Conclusion

Ultimately, the study’s findings have led to the creation and launch of the Tourism Variational Recurrent Neural Network (TourVaRNN). This tool improves economic effect forecasts and gives marketers helpful campaign information. Using variational neural networks with recurrent connections and variational autoencoders makes TourVaRNN a significant step forward in tourism research through its application. It delivers vital information for successful marketing strategies and tourist management in a global industry that is continually shifting. These insights are provided by precisely forecasting economic effects and visitor behaviors. The robust performance measures that characterize the model highlight its practical relevance in improving budget allocation and improving procedures for making choices within the industry. TourVaRNN’s improved segmentation efficiency of 15.7 percent allows for more targeted marketing, increasing engagement with visitors and income. Decisions may be made in real-time, improving operational efficiency in tourism management, thanks to a 17.5% reduction in inference time (to 40 ms). The most efficient use of funds is guaranteed by a 13.4% rise in budget allocation utilization, leading to maximum economic benefits. These innovations support sustainable economic growth, better visitor experiences, and data-driven tourist policy.

### 5.1 Limitations

Although TourVaRNN has many advantages, it also has a few constraints, such as the possibility of being sensitive to data quality since it varies in different tourism regions. The interpretability of the model, its scalability to more enormous datasets, and integration with real-time data sources could be the focus of future developments. These enhancements would strive to improve prediction capabilities and responsiveness in tourism contexts that are constantly changing.

### 5.2 Applications

The research directly improves marketing strategies in the tourism industry by providing actionable insights through precise visitor behavior forecasts, enabling targeted campaigns and efficient budget allocation.

### 5.3 Future research scope

Furthermore, there is the possibility of expanding the use of TourVaRNN beyond economic impact prediction and visitor behavior prediction to encompass more significant areas of tourism sustainability, environmental impact evaluation, and the optimization of individualized tourist experiences. The research scope is extended to dynamically monitor the environment using real-time data from sensors and social media, create multi-task learning models to forecast economic and environmental repercussions, engage with environmental agencies to enhance datasets, implement sustainable tourist policies, and more. These updates will make TourVaRNN more flexible and encourage ecotourism.

TourVaRNN has the potential not just to contribute to more knowledgeable decisions in the tourist management industry but also to play a crucial part in creating tourism practices that are both environmentally responsible and technology-driven on a global scale.
